# Evaluation of the therapeutic efficacy of *Vitex agnus-castus* extract on cisplatin-induced hematotoxicity in female Wistar rats

**DOI:** 10.14202/vetworld.2023.2186-2191

**Published:** 2023-11-01

**Authors:** Aparna Tripathy, Archana Parampalli Raghavendra, Babi Dutta, Sudarshan Surendran

**Affiliations:** 1 Division of Physiology, Department of Basic Medical Sciences, MAHE, Manipal, Karnataka, India; 2 Division of Biochemistry, Department of Basic Medical Sciences, MAHE, Manipal, Karnataka, India; 3 Department of Clinical Anatomy and Medical Imaging, American University of Antigua College of Medicine, Antigua

**Keywords:** cisplatin, hematotoxicity, rats, *Vitex agnus-castus*

## Abstract

**Background and Aim:**

Cisplatin (CP) is a preferred drug for cancer treatment but it has dose-dependent side effects. *Vitex agnus-castus* (VAC) berry extract has antioxidant, free-radical scavenging, and anti-inflammatory activities. This study explored the mitigating effects of VAC extract (VACE) on acute hematotoxicity induced by CP in female Wistar rats.

**Materials and Methods:**

Female Wistar rats (n = 30) were randomly divided into five groups (n = 6/group). The normal control (NC) group received no treatment. The CP control group received CP (7 mg/kg.b.w. ip, single dose) and the drug control group (VACE-650) received VACE (650 mg/kg b.w. oral, daily) for 7 days. Both groups received a single dose of CP (7 mg/kg b.w. ip), followed by 350 and 650 mg/kg.b.w. of VACE daily orally (CPVACE-350 and CPVACE-650 groups, respectively) for 7 days.

**Results:**

After a single dose of CP (7 mg/kg b.w.), the red blood cells (RBC), hematocrit (HCT), white blood cells (WBC), and platelets significantly decreased. In the VAC-350 group, the reduction in total WBC count was less than that in the VAC-650 group on the 3^rd^ day. The RBC and HCT values of the VACE groups were better than that of the CP control, but the VACE-350 treatment group showed significant improvement only on the 3^rd^ day.

**Conclusion:**

Our findings showed that VACE can mitigate CP-induced damage to peripheral blood cells at lower doses.

## Introduction

Cisplatin (CP) is a platinum-based anti-tumor agent and a preferred drug for treating various solid tumors and malignancies, such as head and neck, testis, ovary, and breast cancers [1–3]. Cisplatin acts on cells by impairing the basic cellular repair and regeneration processes by cross-linking with DNA and inducing protein adducts, which induce oxidative stress and cell apoptosis [4–6]. Although CP is preferred for treating various solid tumors, its use is restricted by its dose-dependent side effects, including nephrotoxicity, neutropenia, anaphylactic reactions, hemolytic anemia, and myelosuppression [7–12]. As CP can act on both cancer and normal cells, understanding the mechanism by which it induces oxidative stress in normal cells can reduce the associated side effects. Many compounds, such as resveratrol [9], quercetin [13], curcumin [14], and L-carnitine [15], have been shown to ameliorate CP toxicity in normal tissues. Several plant extracts, including *Ginkgo biloba*, *Schisandra sphenanthera*, *Ficus religiosa*, *Stevia rebaudiana*, and *Citrullus colocynthis*, have also been used in this regard due to their non-toxic nature. Plant compounds, including flavonoids, essential oils, saponins, alkaloids, and polysaccharides, can act synergistically to limit oxidative stress [16]. *Vitex agnus-castus* (VAC) is a deciduous shrub found in the Mediterranean regions of Europe and Central Asia, and its fruits contain major active constituents, such as agnoside, oleic acid, and casticin [17]. The VAC fruits have been used in herbal medicine as dietary supplements for treating mastalgia, corpus luteum insufficiency, irregular lactation, and menstrual cycle abnormalities [18]. Studies have shown that the hydro-alcoholic extract of these fruits has anti-tumor and anti-proliferative activity against prostate cancer and Michigan Cancer Foundation (MCF)–7 breast cancer cells, cervical carcinoma (SKG-3a), and gastric signet ring carcinoma (KATO-III) [19–21]. *Vitex agnus-castus* extract (VACE) has also been shown to have nephroprotective effects in the D-galactose-induced age-related changes in kidneys [22].

Despite the studies supporting the role of VAC in protecting normal cells during cancer, its protective function against the action of CP in blood has not yet been investigated. Studies have shown that the VAC berries have anti-inflammatory, antioxidant, and free-radical scavenging activity, which are beneficial in oxidative damage-induced kidney disease [17, 19, 23]. Hence, this extract may be used to minimize the side effects of CP on normal cells.

As the hemopoietic system is a highly sensitive system for toxicity studies, we aimed to investigate the mitigating effect of VACE on acute hematotoxicity (decrease in red blood cells [RBCs], white blood cells [WBCs], and platelet count) induced by CP in female Wistar rats.

## Materials and Methods

### Ethical approval

This study was approved by the Institutional Animal Ethics Committee of Manipal Academy of Higher Education (IAEC/KMC/98/2019).

### Study period and location

This study was conducted from January to April 2021 at Central Animal Research Facility of Manipal Academy of Higher Education, Manipal, India.

### Chemicals and drugs

Commercially available CP injection (10 mg/10 mL) was obtained from GLS Pharma Limited, Hyderabad, Telangana, India. Based on the animal’s body weight, the volume of the injection was calculated and an undiluted CP injection (7 mg/kg bw) was administered.

### *Vitex agnus-castus* extract

We obtained the VACE from Green Heaven India, Nagpur, Maharashtra, India. We prepared a stock solution by dissolving 200 mg of the extract in 1 mL of deionized water. The required amount of VACE was dissolved in deionized water at 25°C–27°C and mixed using a magnetic stirrer for 5 min until the solution became clear. After incubating the solution for 24 h with periodic stirring, it was filtered through Whatman filter paper 1. No precipitate or suspension was observed on the filter paper and the final solution (200 mg/mL) was stored at 4°C.

During the experiment, the animals were weighed daily. The volume of the extract was calculated and administered orally based on the specified doses at 350 and 650 mg/kg b.w.

### Experimental animals

We randomly selected 30 female Wistar rats (weighing 180–250 g) and divided them into five groups (n = 6/group). We selected female rats because there are many studies present on male animals, survival rate of female rats was better than male rats. These animals were kept in standard cages covered with sterilized husks. They were maintained under standard conditions at a temperature range of 25°C–27°C, a 12-h light/dark cycle, and continuous access to a regular rat pellet diet and drinking water. Three animals were housed in each cage to prevent overcrowding, and they were acclimatized to the laboratory environment for 7 days before the experiment.

### Experimental design (Figure-1)

The normal control (NC) group was left untreated. The CP control group received CP (7 mg/kg.b.w. ip, single dose) and the drug control group (VACE-650) received VACE (650 mg/kg b.w. oral, daily) for 7 days. Then, both groups (CPVACE-350 and CPVACE-650) received a single CP dose (7 mg/kg b.w. ip), followed by daily oral administration with 350 and 650 mg/kg b.w. of VAC (CPVACE-350 and CPVACE-650 groups, respectively) for 7 days. The blood samples were collected using ethylenediamine tetraacetic acid tubes. Before (day 0) and 72 and 144 h after CP/VAC administration (days 3 and 6), we performed a complete blood count individually using a veterinary blood cell counter (PCE-210VET, ERMA INC, Tokyo). All the animals were euthanized with a high dose of phenobarbital at the end of the experiment.

**Figure-1:**
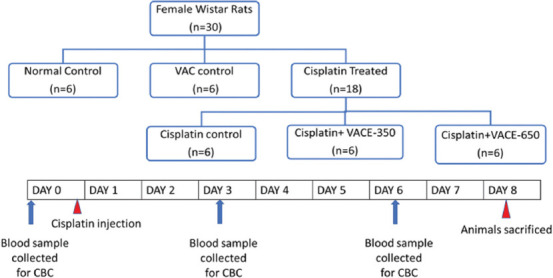
Schematic representation of cisplatin and VACE treatment and sample collection time points (VACE-350: *Vitex agnus-castus* extract 350 mg/kg.b.w, VACE-650: *Vitex agnus-castus* extract 650 mg/kg.b.w). VAC=*Vitex agnus-castus*, CBC=Complete blood count.

### Statistical analysis

The data obtained were analyzed using the Statistical Package for the Social Sciences (version.22.0), and normally distributed data are expressed as mean ± standard deviation. Data were analyzed by repeated measure analysis of variance (ANOVA) with *post hoc* Bonferroni test for comparing the changes within groups. One-way ANOVA with *post hoc* Tukey test was used to compare different groups. p < 0.05 was considered statistically significant.

## Results

### Comparison of the blood cell counts within the group at different time points

#### Effect on WBCs (Figure-2)

The WBC count was decreased after CP (7 mg/kg b.w.) administration on the 3^rd^ day but was restored to near normal by the 6^th^ day. In the VACE (650 mg/kg b.w.) group, the WBC count increased on the 3^rd^ day and decreased insignificantly by the 6^th^. In both CPVACE-350 and CPVACE-650 groups, a decrease in WBC count was observed on the 3^rd^ day, though not statistically significant, and was restored to normal by the 6^th^ day.

**Figure-2:**
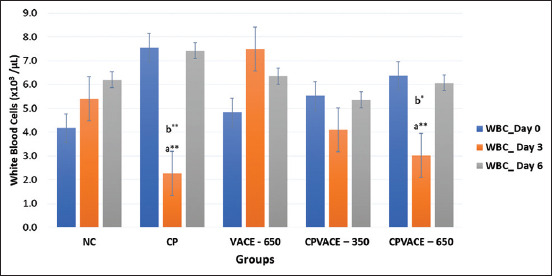
Changes in the mean white blood cells in controls and experimental groups at different time points (Mean values and error bars of ± 2 SE) (n = 6). *p < 0.05, **p < 0.01 comparison with day 0/normal control, ^#^p < 0.05 comparison to cisplatin control, a – Repeated measure analysis of variance values in individual groups on different time points. b – One-way analysis of variance values comparing among different groups. SE=Standard error, NC=Normal control, CP=Cisplatin, VACE=*Vitex agnus-castus* extract.

#### Effect on RBCs and hematocrit (HCT) (Figures-3 and 4)

After CP (7 mg/kg b.w.) administration, a statistically significant reduction in the RBC and HCT counts were observed on days 3 and 6 compared to day 0. In the VACE (650 mg/kg b.w.) group, the RBC count and HCT% gradually but significantly decreased on days 3 and 6 compared to that on day 0. In the CPVACE-350 group, a reduction in RBC and HCT was observed on day 3, though not statistically significant. However, the RBC count and HCT on day 6 were significantly lower than that on day 0. In the CPVACE-650 group, the RBC count and HCT% decreased significantly on days 3 and 6 compared to day 0. We observed that, on day 3, the reduction in the RBC count and HCT% in the CPVACE-350 group was lower than that of the CPVACE-650 group.

**Figure-3:**
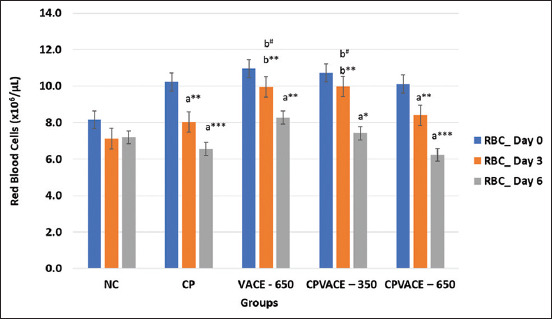
Changes in the mean red blood cells in controls and experimental groups at different time points (Mean values and error bars of ± 2 SE) (n = 6). **p < 0.01, ***p < 0.001 comparison with day 0/normal control. ^#^p < 0.05 comparison to cisplatin control. a – Repeated measure analysis of variance values in individual groups on different time points. b – One-way analysis of variance values comparing among different groups. SE=Standard error, NC=Normal control, CP=Cisplatin, VACE=*Vitex agnus-castus* extract.

**Figure-4:**
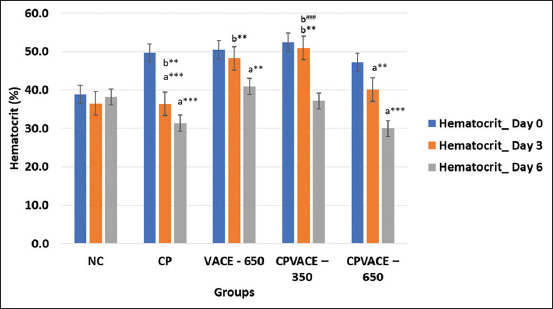
Changes in the mean hematocrit values (%) in controls and experimental groups at different time points (Mean values and error bars of ± 2 SE) (n = 6). **p < 0.01, ***p < 0.001 comparison with day 0/normal control. ^###^p < 0.001 comparison to cisplatin control. a – Repeated measure analysis of variance values in individual groups on different time points. b – One-way analysis of variance values comparing among different groups. SE=Standard error, NC=Normal control, CP=Cisplatin, VACE=*Vitex agnus-castus* extract.

#### Effect on platelets (Figure-5)

The platelet count was increased after CP (7 mg/kg b.w.) administration on day 3 but decreased by day 6, though not statistically significant. Although the platelet count increased on both days 3 and 6 in the VACE-650 group, the increase was statistically significant only on day 6 compared to day 0. In both CPVACE-350 and CPVACE-650 groups, the platelet count was increased on day 3, though not statistically significant.

**Figure-5:**
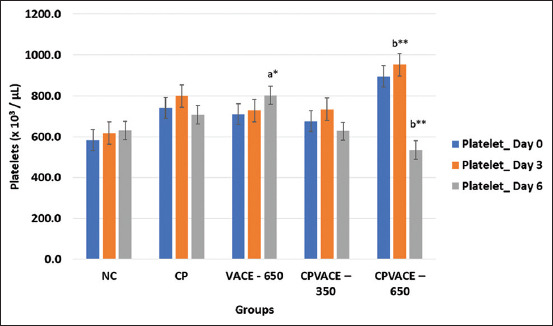
Changes in the mean platelet values in controls and experimental groups at different time points (mean values and error bars of ± 2 SE) (n = 6). *p < 0.05, **p < 0.01 comparison with day 0/normal control. a – Repeated measure analysis of variance values in individual groups on different time points. b – One-way analysis of variance values comparing among different groups. SE=Standard error, NC=Normal control, CP=Cisplatin, VACE=*Vitex agnus-castus* extract.

### Comparison of the blood cell counts across the groups

#### Effect on WBCs (Figure-2)

In the CP control group, the WBC count on day 3 was significantly decreased than the NC group. The WBC count was significantly increased in the VACE group compared to both NC and CP control groups. However, while there was no significant change in both groups on day 3 compared to NC, the WBC count returned to near normal on day 6.

#### Effect on RBC (Figure-3)

Treatment with VACE increased the RBC count compared to NC and CP control groups. While the RBC count was significantly higher in the CPVACE-350 group on day 3, there was negligible change in the CPVACE-650 group compared to both control groups.

#### Effect on HCT (Figure-4)

There was a statistically significant decrease in the HCT value on day 3 in the CP control group compared to the NC group. However, the HCT values were higher in the VACE group compared to the NC and CP groups. On day 3, the HCT value was significantly higher in the CPVACE-350 group, whereas there was no significant change in that of the CPVACE-650 group compared to both control groups.

#### Effect on platelets (Figure-5)

In the CPVACE-650 group, the platelet count was significantly increased on day 3 but significantly decreased on day 6 compared to the NC group.

## Discussion

Administration of a single dose of CP (7 mg/kg b.w.) significantly decreased the RBC, WBC, platelet count, and HCT value, which reflects bone marrow damage. This observation was consistent with the previous studies by Olas and Wachowicz [9], and Sushmitha *et al*. [10], which suggested that CP acts on the proliferating cells in the bone marrow, disrupting hematopoiesis (myelosuppression). Cisplatin also causes kidney damage, decreases renal erythropoietin, and inhibits erythropoiesis (renal anemia) [24]. Cisplatin modulates the intracellular antioxidant (glutathione) levels required to maintain the integrity of RBCs’ membranes, increasing the osmotic fragility of RBCs, which causes hemolytic anemia [25, 26]. Bone marrow damage and osmotic fragility can cause a reduction in the total RBC count, which was also reflected in the HCT value [27].

Gas chromatography-mass spectrometry analysis of the VAC berry extract showed that it contained a high quantity of oleic and linoleic acids (data not shown). Both these fatty acids possess intrinsic anti-inflammatory properties. Oleic acid also has antioxidant activity [28]. A study by Limbkar *et al*. [29] also showed that linoleic acid can stimulate hematopoietic stem and progenitor cells.

In our study, we observed that the WBC count significantly decreased 72 h after CP treatment, which is consistent with the previous studies by Olas and Wachowicz [9], Sushmitha *et al*. [10], and Khynriam and Prasad [26]. We also observed that the total WBC count returned to normal (i.e., pre-CP levels) around day 6 (144 h) after CP treatment, which aligns with the previous study by Nowrousian and Schmidt [30]. This increase in the WBCs on day 6 might be due to infection and inflammation during CP administration and CP metabolism in the rats [9, 31]. While similar effects were observed in both VAC-treated groups, the extent of decrease in the total WBCs on day 3 in the VAC-350 group was lesser than that in the VAC-650 group, indicating that treatment with 350 mg/kg b.w. of VAC might shield the blood cells from chemotherapy insult.

We observed a decrease in the total RBC count and HCT in all the CP-treated groups as CP causes bone marrow damage and disrupts RBC membrane integrity [32]. All the VAC-treated groups showed improvement in both RBC and HCT compared to CP, but significant improvement was seen only on day 3 in the VACE- 350 group.

We observed an increase in the platelet count (thrombocytosis) 72 h after CP administration, followed by a decrease on day 6 (144 h). The initial increase in the platelet count might be due to autonomous overproduction or reactive thrombocytosis due to hemolysis, infections, or inflammation. Cisplatin can also directly cause thrombocytosis by exacerbating iron deficiency, which favors thrombocytosis [33–36]. We observed erythrocytopenia and thrombocytosis on day 3 after administration of CP in all CP-treated groups. Although these differences in the platelet count were not statistically significant, these observations could be clinically important.

Our findings showed that treatment with VACE-350 showed a better protective effect on blood cells than with VACE-650. However, the reason behind this difference is unknown as there is limited information regarding the actions of all the components in the VACE. Previous studies [28, 37] have shown that the fatty acids in the VACE have both anti-inflammatory and antioxidant activities. Contrarily, some studies have also suggested that both oleic and linoleic acids can cause cytotoxicity depending on the concentration and period of exposure, which has not been established yet [38]. Moreover, linoleic acid is also known to be highly vulnerable to oxidative stress, leading to cell damage [39]. In this study, we found that CP causes oxidative stress, which might affect the stability of linoleic acid. Both oleic and linoleic acids might act as antioxidants at lower doses, but they are more likely to be pro-oxidants at higher doses.

## Conclusion

Treatment with VACE could mitigate CP-induced damage to peripheral blood cells at lower doses. Additional clinical studies are required to derive an appropriate dose for humans to alleviate CP damage and improve the quality of life of CP-treated patients.

## Authors’ Contributions

AT, APR, and SS: Study conceptualization and study design. AT and BD: Performed experiments and investigations. AT, BD, and SS: Statistical analysis. AT and APR: Initial draft of the manuscript. AT, APR, BD, and SS: Review and editing of the manuscript. SS and APR: Overall project supervision. All authors have read, reviewed, and approved the final manuscript.
